# Does Beetroot Supplementation Improve Performance in Combat Sports Athletes? A Systematic Review of Randomized Controlled Trials

**DOI:** 10.3390/nu15020398

**Published:** 2023-01-12

**Authors:** Slaheddine Delleli, Ibrahim Ouergui, Hamdi Messaoudi, Khaled Trabelsi, Jordan M. Glenn, Achraf Ammar, Hamdi Chtourou

**Affiliations:** 1 Research Unit, Physical Activity, Sport and Health, UR18JS01, National Observatory of Sport, Tunis 1003, Tunisia; 2 High Institute of Sport and Physical Education of Sfax, University of Sfax, Sfax 3000, Tunisia; 3 Research Unit: Sport Sciences, Health and Movement, UR22JS01, University of Jendouba, Kef 7100, Tunisia; 4 High Institute of Sport and Physical Education of Kef, University of Jendouba, Kef 7100, Tunisia; 5 Research Laboratory: Education, Motricity, Sport and Health, EM2S, LR19JS01, University of Sfax, Sfax 3000, Tunisia; 6 Department of Health, Exercise Science Research Center Human Performance and Recreation, University of Arkansas, Fayetteville, AR 72701, USA; 7 Department of Training and Movement Science, Institute of Sport Science, Johannes Gutenberg-University Mainz, 55122 Mainz, Germany; 8 Interdisciplinary Laboratory in Neurosciences, Physiology and Psychology: Physical Activity, Health and Learning (LINP2), UFR STAPS (Faculty of Sport Sciences), UPL, Paris Nanterre University, 92000 Nanterre, France

**Keywords:** nitrate, combat sports, ergogenic aid, performance

## Abstract

While studies on dietary nitrate (NO_3_^−^) supplementation and its impact on combat sports performance are increasing, finite conclusions from currently available investigations remain unclear. Thus, the present systematic review examined the acute and chronic ergogenic effect(s) of dietary nitrate intake from beetroot on different aspects of combat sports performance. A systematic search for randomized placebo-controlled studies investigating the effects of beetroot supplementation on combat sports outcomes was performed through Scopus, PubMed/MEDLINE, Web of Science, Scielo, Sport Discus, and Cochrane Library databases up to 2 January 2023. The different terms related to beetroot and to combat sports were connected in the search strategies using the Boolean operators ‘AND’ and ‘OR’. A total of nine studies with good methodological quality (based on the Cochrane risk of bias tool) fulfilled the inclusion criteria. Seven studies used an acute supplementation strategy, while the other two studies utilized chronic supplementation. Findings showed beetroot intake may be an effective tool to improve oxidative metabolism and muscle force production (i.e., isokinetic and isometric) in combat sports athletes. However, these effects may depend on the population, intake duration, muscle group activated, and exercise type. Future studies are required to (1) understand the effects on female athletes and (2) elucidate the impacts of dosing protocols and specific exercise modalities for enhancing combat sports performance.

## 1. Introduction

During the high-level athletic competition, victory is often decided by nuanced differences [1], which affect the likelihood of winning [2]. As a result, sports scientists, athletes, and coaches are constantly evaluating innovative strategies to enhance performance [2]. Ergogenic aids are one such method that can be effective in improving the physiological and physical attributes of athletes [3,4] and competitive athletes frequently use nutritional supplements to improve their performance [5]. The high amount of inorganic nitrate (NO_3_^−^) found in beetroot has made it a popular subject for research on nutritional supplements [6]. It is well recognized that NO_3_^−^supplementation may increase exercise tolerance in healthy young men following a single dose or multiday consumption [7,8]. The effects of dietary NO_3_^−^intake are attributed to increases in nitric oxide (NO). When consumed, facultative anaerobic bacteria on the tongue’s dorsal surface convert NO_3_^−^ into bioactive nitrite (NO_2_^−^) [9]. Through the action of stomach acids, this NO_2_^−^is then partially converted to NO by lucking oxygen [10]. Once in the systemic circulation, NO is absorbed in the heart, skeletal muscle, and blood vessels [11]. Acidic and hypoxic circumstances, often present during exercise, aid in the conversion of NO_2_^−^ to NO [4,12]. During skeletal muscle contractions, NO acts as a major vasodilator, promoting physiological reactions and affecting oxygen kinetics [7]. Especially, increased NO availability may benefit oxygen and nutrient delivery by boosting blood flow to the working muscle [7]. In terms of metabolic function, NO is believed to improve glucose absorption and mitochondrial efficiency in muscle, thereby reducing the adenosine-triphosphate (ATP) cost of muscle contractions and oxygen expense [4,13]. Through this pathway, beetroot intake has been reported to enhance performance during endurance [14], explosive [15], and intermittent [16] activities. However, the impact of sports supplementation on performance can vary greatly based on the sport modality and prescribed dose [17]. In fact, when compared to placebo, performance with NO_3_^−^ supplementation does not improve in over 70% of trials investigating possible performance-enhancing benefits [18].

In combat sports, repeated high-intensity efforts during competition require significant contributions from both oxidative and non-oxidative energy systems. According to Campos et al. [19], oxidative metabolism accounts for approximately 66% of energy output during taekwondo combat simulation. As such, increasing oxidative capacity is crucial for high performance in combat sports [20], as it allows an athlete to better recover between high-intensity bouts and during low-intensity actions [20]. Given that beetroot supplementation may increase oxygen and substrate delivery to the active skeletal muscle and potentially improve exercise performance [4,13], it is important to apply these findings to sport-specific areas, such as combat sports. In fact, the development of a physical and physiological profile suitable to the requirements of each discipline determines performance in both striking and grappling contests [21]. However, the repetitive high intensity nature of combat sports leads to high levels of peripheral fatigue, impairing performance [22,23]. Since NO is purported to delay fatigue and increase exercise tolerance, which are essential factors when exercising at high intensities [7], NO_3_^−^ intake could have a significant impact on combat sports performance [24]. Prior research reports beetroot supplementation can speed up recovery through improved oxygen delivery during rest intervals between high-intensity actions [16]. As such, it can be hypothesized that the beetroot’s ergogenic effect would be greatest during intermittent exercise (e.g., combat sports).If the conversion of nitrate to NO is enhanced under conditions of hypoxia and raised acidity [4,12], then it may be most effective during these kinds of moments. However, like in other sports modalities, there are inconsistent findings regarding beetroot’s benefits in combat sports [25,26]. Beetroot supplementation’s ergogenic effectiveness is likely determined by a multitude of descriptive factors including: cardiorespiratory fitness, dose and timing of supplementation [27], work-to-rest ratios [26], environmental factors (e.g., hypoxia) [28], biological sex [29], and inter-individual variability in pharmaco-dynamics and dose-response relationships [27]. Therefore, the ergogenic effects of beetroot supplementation may vary within and between sport-specific situations [16]. 

Beetroot effects have been found to be moderated by sport nature and inter-individual variability [18,27]; however, previous reviews [12,16] have included individuals with a variety of traits and training backgrounds. Since this resulted in inconsistent findings, the decision was to restrict this review to athletes only competing in combat sports. As a result, this paper aimed to review and investigate the scientific knowledge about the effect of nitrate derived from beetroot intake on combat sports outcomes. Likewise, this systematic review will to offer possible explanations for efficacy discrepancies, while highlighting usefulness and providing perspectives and recommendations that may aid future investigations.

## 2. Methods

This is a systematic review of randomized controlled studies with a priori registered protocol on the Open Science Framework (OSF) “https://doi.org/10.17605/OSF.IO/85SRU (accessed on 03 October 2022)”. The systematic review was conducted following the 2020 Preferred Reporting Items for Systematic Reviews and Meta-Analyses (PRISMA) [30] and the PRISMA implemented in Exercise, Rehabilitation, Sports medicine and SporTs science (PERSiST) guidelines [31].

### 2.1. Search Strategy

PubMed/MEDLINE, Scopus, Web of Science, Sport Discus, Scielo and Cochrane Library were searched without date limits or filters. The search was performed up through 2 January 2023 connecting different terms related to combat sports with others related to beetroot. Terms were combined using the Boolean operators ‘AND’ and ‘OR’ with medical subject heading (MeSH) terms and truncations (*) were used appropriately. The search strategy on each database was presented in Table 1.

To minimize the risk of missing relevant manuscripts, a complementary search was carried out to browse the reference lists of the included research and associated review papers [32]. No language restriction was used in the search [33]. The “Endnote 20” software (Camelot UK Bidco Limited-Clarivate, London, UK) was employed to assess the search results. 

### 2.2. Inclusion and Exclusion Criteria

Inclusion criteria were applied following the PICOS (Participants, intervention, comparators, outcomes, and study design) model. Articles were included if they were conducted on combat sports athletes who utilized beetroot supplementation as an intervention in any form or dose. Only placebo-controlled trials were retained. Finally, studies including any physical, physiological, cognitive or psychophysical measurement using a double-blind or single-blind design were included. These eligibility criteria resulted in the exclusion of studies that were not associated with the established PICOS criteria. Additionally, books, citations, trial registry records, conference proceedings, systematic reviews, and narrative reviews were excluded.

### 2.3. Selection Process

Before being taken into consideration for inclusion, the retrieved articles were first checked for duplication using the software “Endnote 20” (Camelot UK Bidco Limited-Clarivate, London, UK). After duplicates were removed, all pertinent articles’ titles were reviewed, and then the remaining articles’ abstracts and fully published articles were examined. The procedure was independently performed by two reviewers. Any disagreements were settled by consensus.

### 2.4. Data Extraction 

For all studies meeting inclusion criteria, data were summarized in a spreadsheet using Microsoft Excel. A piloted data extraction form with the following items: author(s), year of publication, study design, sample size and combat sport name, timing after beetroot intake, beetroot dose, the form of beetroot supplement (i.e., capsule, gel, juice), the outcomes measured, and the main results were used. For the main results, all data concerning the effect of beetroot supplementation on physical performance, perceived exertion and physiological response during or/and following exercise were extracted from the research papers. Participant characteristics in each study were also extracted. Data reporting sex (males; females), age (≤18 years: adolescent; >18 years; adult athlete), experience (<five years: amateur; ≥five years: elite) of athletes, as well as their training regime and country were recorded for analyses. The data extraction process was double-checked by a second reviewer to avoid any selection bias and data extraction flaws [34].

### 2.5. Risk of Bias Assessment

The risk of bias was assessed with the Cochrane risk of bias tool [35]. Two authors assessed the methodological quality independently, with discussion and consensus over any observed differences.

## 3. Results

### 3.1. Search Results

**Figure 1:**
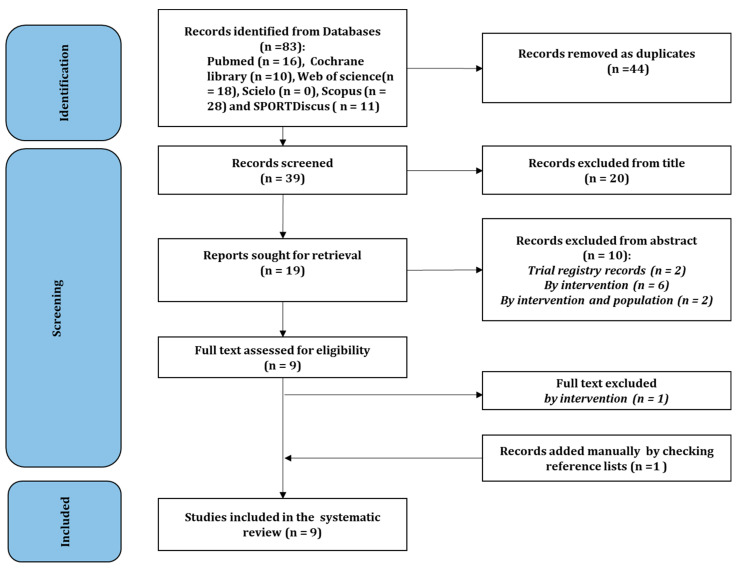
Flow diagram of the search process.

The flow diagram (Figure 1) represents the search process. A total of 83 records were found via the initial search. Among these, 44 records were excluded as duplicates, with 39 records remaining. After screening the titles, 20 were removed. Among the 19 remaining records, 10 were removed after checking the abstract and nine reports remained and were assessed for eligibility. By applying the eligibility criteria, one full-text article was excluded. After conducting an additional search, one article was identified as a potentially relevant study, resulting in a total of nine studies included in this systematic review. The list of excluded studies in each level is presented in the Supplementary Table S1.

### 3.2. Population Characteristics

The characteristics of athletes recruited within studies are presented in Table 2.

The nine reviewed studies included a total of 92 combat sports athletes. From the grappling modality, 42 athletes were recruited, including 16 Greco-Roman wrestlers [36,37], and 12 Brazilian jiu-jitsu athletes [38]. From striking sports, 40 athletes were recruited, including 32 taekwondo athletes [25,26,40] and eight boxers [42]. In addition, 10 athletes were recruited from MMA [39], whereas the background of the remaining 14 athletes was not identified [41].

Eight studies recruited male athletes (i.e., 82 athletes), and one study [39] (i.e., 10 athletes) did not identify the participants’ sex. Regarding age, the recruited athletes were young adults (i.e., >18 yrs), with an age range from 19.2 (1.6) to 29.92 (8.51) yrs. For nationality, three studies (i.e., 24 athletes) were conducted within Turkish athletes [36,37,42], four studies (i.e., 48 athletes) with Brazilian athletes [25,38,39,41], and two studies (i.e., 20 athletes) on Iranian athletes [26,40].

### 3.3. Study Characteristics 

Studies’ characteristics are presented in Table 3.

The nine studies included in this systematic review comprised three trials from grappling, four trials from striking combat sports, one trial from mixed martial arts (MMA), and one study which did not specify the athletes’ background.

Of the included studies, beetroot was administered via an absolute dose in eight trials and via a relative dose (i.e., 2 g/kg of body mass) in a single trial [42]. Administration via capsule of 1 g of beetroot extract was used in one trial [25]. A nutritional gel of 100 g of beetroot was used in two trials [38,41]. For studies administered in juice format, the doses were 60 mL [26], 120 mL [26], 140 mL [36,37,40], and 400 mL [39]. For the timing between beetroot supplementation and the subsequent performance testing, the waiting time ranged from 2 to 2.5 h. Among the nine studies, two tested the chronic effects of beetroot supplementation for eight [38] and six [40] days, whereas, in the remaining other studies [25,26,36,37,39,41,42], acute effects were investigated.

To investigate the effect of beetroot supplementation on physical performance, several testing procedures were utilized. For the upper body, the handgrip isometric exercise was used [38,41]. For the lower body, the countermovement jump (CMJ) [26], the repeated sprint test (20 all-out 6 s sprints) [39], the 30-s all-out Wingate test [42], and the isokinetic knee extensions [36,40] were performed. Moreover, to assess the impact of beetroot supplementation after a fatiguing strength exercise (i.e., Maximal contractions knee extension and flexion at the speed of 60° s^−1^ (5 times) and 180° s^−1^ (15 times)), dynamic and static balance were tested [37]. To examine the effect of beetroot supplementation on specific combat sports’ performance, specific physical fitness tests were used. Specifically, the progressive specific taekwondo test (PSTT) [25,26] and the multiple frequency speed of kick test (FSKT-mult) [26] were used for taekwondo practitioners.

To assess the associated physiological responses to beetroot supplementation, heart rate (HR) [26,40,42], blood lactate concentration [bLa-] [25,26,42], maximum oxygen (O_2_) consumption (VO_2max_) [25], blood volume [38,41], blood pressure [40], and muscle oxygenation [38,41] were measured. For cognitive assessment, the Stroop test was used [26]. In addition, to measure subjectively perceived fatigue, the rating of perceived exertion(RPE) 6–20 Borg scale was used [26,40].

### 3.4. Methodological Quality 

**Figure 2:**
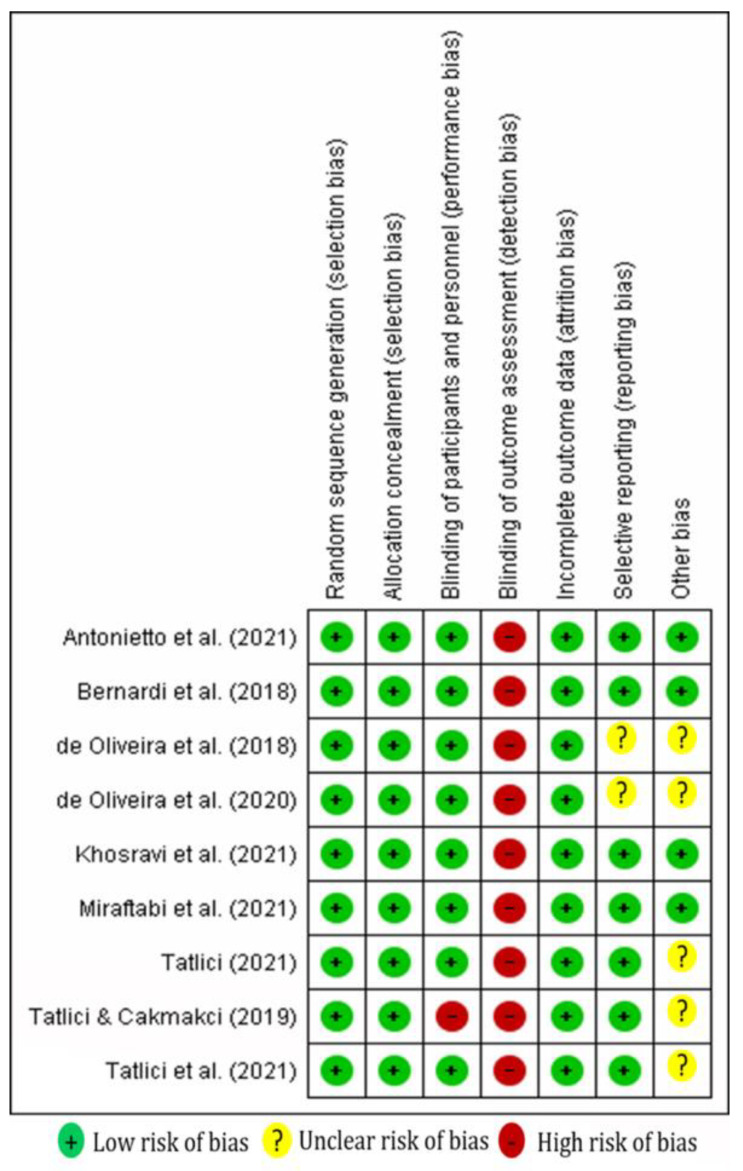
Risk of bias summary: Review authors’ judgments about each risk of bias item for each included study [25,26,36,37,38,39,40,41,42].

The nine included studies showed good methodological quality. The study conducted by Tatlici and Cakmakci [42] failed to satisfy the ‘‘assessor blinding and outcome blinding criterions”, whereas in the other studies, the double blinding design was satisfied. The risk of bias assessment of the included studies is presented in Figure 2.

### 3.5. Studies’ Main Results

#### 3.5.1. Isometric and Isokinetic Strength

In the present systematic review, two of the included studies assessed the effect of beetroot intake on isometric strength [38,41], and two evaluated isokinetic strength [36,40]. The acute (i.e., 2–2.5 h before physical exercise, a dose of 140 mL) beetroot intake contributed to improvements in isokinetic strength [36], whereas chronic (i.e., 140 of beetroot juice/day, for a minimum of six-eight days) intake improved isokinetic performance [40] and prevented isometric strength decline after fatiguing exercise [41]. Regarding the acute effects of beetroot supplementation, improvements were recorded in the isokinetic strength of the shoulder (15.06% for internal and 13.39% for external torque) [36], and average knee strength (9.57% for extension and 12.94% for flexion) [36]. Regarding chronic effects, two investigations examined the effects of six [40] and eight days [38] of supplementation with beetroot juice and a beetroot-based nutritional gel, respectively. Using isokinetic exercise, Khosravi et al. [40] reported an increase in knee extensor peak torque by ~6% at angular velocities of 180 in the dominant leg and 1.5% at 360°/s in the non-dominant leg. Using isometric exercise, de Oliveira et al. [38] reported significant reductions in the delta of muscle voluntary contraction decline after handgrip isometric exercise following 2 h of supplementation with 100 g of beetroot-based nutritional gel.

#### 3.5.2. Power Performance

Regarding beetroot supplementation’s impact on power exercise, two studies investigated the acute effect of beetroot juice on countermovement jump performance (CMJ) [26] and the 30 s Wingate test [42]. Trials revealed unchanged jump height in taekwondo athletes 2.5 h after the intake of 400 and 80 mg of NO_3_^−^ [26] and a decrease in peak power and mean power output in boxers 2.5 h after the ingestion of 2 mL/kg of beetroot juice [42].

#### 3.5.3. Intermittent Performance

The effect of beetroot intake on intermittent performance was investigated in two trials using 20 all-out 6 s sprints interspersed with 24 s recovery and the specific FSKT-mult [26,39]. Results revealed the supplement had no acute discernible impact on taekwondo or MMA performance after the ingestion of 400 and 800 mg of NO_3_^−^ [26] or 9.3 mmol of NO_3_^−^ [39] using 2 and 2.5 h of rest, respectively.

#### 3.5.4. Endurance Performance

In the present systematic review, two studies [25,26] investigated the effects of beetroot supplementation on endurance performance using the PSTT. Even with the same testing protocol and sport modality (i.e., taekwondo), the two studies reported inconsistent results. While Antonietto et al. [25] found 1 g of acute beetroot extract increased peak oxygen uptake (VO_2Peak_) by 11%, and absolute VO_2max_ at the ventilatory threshold by 15.6% during the PSTT, Miraftabi et al. [26] reported no significant improvements following 60 mL and 120 mL of beetroot juice intake.

#### 3.5.5. Balance, Perceived Exertion, and Cognitive Function

In the present systematic review, one study [37] investigated the acute impact of 140 mL of beetroot juice on the dynamic and static balance of Wrestlers at rest and after strenuous activity. In this study, Tatlici et al. [37] reported an improvement of the stability index of 2.5 h after beetroot intake, pre-and post- the fatiguing stimulus. For perceived exertion, two trials [26,40] assessed the effects of beetroot supplementation on RPE values following isokinetic (i.e., isokinetic knee extensions), explosive (i.e., CMJ), intermittent (i.e., FSKT-mult), and progressive endurance (i.e., PSTT) performance. Consistently, findings from these trials showed indifferent RPE values across conditions [26,40]. Regarding cognitive function, Miraftabi et al. [26] used the Stroop test to evaluate cognitive function in taekwondo athletes. The study found that 400 mg of NO_3_^−^ 2.5 h before testing improved cognitive function in the athletes tested.

## 4. Discussion

The present systematic review aimed to summarize the currently available studies investigating the effects of beetroot supplementation on combat sports outcomes, while providing insights into the observed contradictions among different intervention protocols. Due to the discrepancies in the outcomes evaluated in the reviewed studies, the different variables have been clustered by exercise type for a more comprehensive discussion.

### 4.1. Effects of Beetroot Intake on Isometric and Isokinetic Strength

Beetroot’s ergogenic benefits were apparent in the mitigation of forearm muscle strength’ impairment following a strenuous exercise [41]. This finding supported previous meta-analysis results, where beetroot juice accelerated muscle recovery and reduced muscle soreness following strenuous exercise [43]. These outcomes may be linked to the capacity of nitrate to improve exercise effectiveness and prolong exhaustion by enhancing the efficiency of ATP consumption during muscular contraction thus reducing the force production cost [44,45]. Additionally, earlier research reported beetroot supplementation improved fatigue resistance by boosting calcium release in the muscle and the contractility of fast-twitch muscle fibers [2]. The pumping of calcium into the sarcoplasmic reticulum is well known to use up to 50% of the total ATP turnover, which is one of the energy-intensive processes involved in skeletal muscle contractions [46,47].

Although measuring force production using isokinetic strength assessment is a very accurate procedure [48], there were inconsistent findings regarding supplementation effects on isokinetic strength. Current results from combat sports athletes do not necessarily support previous reports [49]. More specifically, acute dietary NO_3_^−^ingestion did not induce positive benefits on muscle peak torque production in the lower limbs, regardless of the angular velocity imposed [49]. The conflicting results may be associated with the activated muscle groups [36]. The type of muscular contraction may determine the beneficial effect of beetroot supplementation on muscle performance [38]. As such, it has been suggested that NO_3_^−^may be more effective during fast-twitch fibers (i.e., fibers composed of a high percentage of myosin heavy chain (MHC) type IIa and IIx) contractions [50]. Therefore, the percentage of fast MHC distribution may be responsible for the inconsistent effects of beetroot supplementation on muscular strength [36].

### 4.2. Effects of Beetroot Intake on Power Exercise

The lack of performance enhancement during a power exercise recorded in the present systematic review may be attributed to the short duration of the test, as the ergogenic effect of supplementation requires a longer physical stimulation time [25]. For time trials, results from this systematic review could support previous work by McMahon et al. [14]. In fact, according to the exercise protocol used (i.e., time trials, open-ended tests, and graded-exercise tests), McMahon et al. [14] observed that dietary NO_3_^−^ supplementation improved performance only when an open-ended test was used. In this context, Silva et al. [51] found through their meta-analysis that nitrate intake was most effective for exercises between 2 and 10 min in duration. By contrast, they revealed during short-duration exercises (i.e., ≤30 s) such as the 30-s Wingate test nitrate was ineffective [51]. Therefore, nitrate intake appears ineffective for exercises that have a reliance on extra-mitochondrial energy systems [14]. However, using more sport-specific exercises could be an important factor to consider in future investigations on combat sports. 

### 4.3. Effects of Beetroot Intake on Intermittent Exercise

Taking into consideration the intermittent nature of the tasks used, the present systematic review findings did not support those reported by Dominguez et al. [16], who observed that either single or repeated doses increased work output during intermittent exercise. Since NO_3_^−^supplementation efficacy is influenced by the population evaluated, this fact could explain the inconsistent findings [4,52]. For example, basal nitrate synthesis is higher in individuals with high cardio-respiratory fitness and lower in individuals with underlying pathology [53]. Additionally, highly trained subjects are likely to have higher NO synthesis activity, which might render the NO_3_^−^–NO_2_^−^–NO pathway less important for NO production [54]. Therefore, individuals with more training may experience attenuated benefits from NO_3_^−^supplementation [55]. Despite the smaller impact of NO_3_^−^on the performance of well-trained individuals, the effects should not be neglected [1]. Additionally, since fatigue is task-dependent, it may vary depending on exercise type [18]. In fact, the type of muscular contractions and level of muscle activation are primary components in determining fatigue (e.g., concentric vs. eccentric, upper-body vs. lower-body) [56]. For short-duration repeated high-intensity exercises, NO_3_^−^benefits may predominate during the initial bouts of contraction [12]. As such, it was shown power output improved during shorter (6 s) maximal sprints compared to longer (30 s) maximal sprints [57]. Furthermore, a recent meta-analysis [12] reported the peak power output of single maximum sprinting efforts was more likely to be improved through supplementation, but not during repeated maximum sprints. These findings suggest the benefits of dietary NO_3_^−^may be better conferred to contractions at earlier phases of force production. By contrast, data from the included studies revealed no discernible effect of beetroot supplementation during initial exercise bouts, compared to subsequent ones [26,39].

Considering the suggested benefits of beetroot on accelerating recovery between high-intensity subsequent efforts [16], the findings from the included studies were unsupportive. The work volume, combined with short rest periods, resulted in gradual depletion and, eventually, fatigue [16]. Given the high-intensity nature of the activities, the high activation of fast-twitch (i.e., MHC IIa and IIx) led to more exhaustion, when compared to slow-twitch (i.e., MHC I) [58]. Notably, type II muscle fibers primarily support non-oxidative pathways and have a better ability for storing creatine, thus favoring glycolytic metabolism [58]. Although oxidative metabolism dominates during fights, non-oxidative metabolism is most likely the principal source sustaining repeated high-intensity movements [59]. Hence, although they potentiate oxidative phosphorylation, beetroot had no effects on glycolytic energy metabolism and was unable to reduce acidosis [16]. The failure to elicit beneficial effects from acute NO_3_^−^dosing suggests the need forlonger, chronic dosing protocols with respect to enhancing intermittent performance. Due to the temporal frame provided for structural and functional alterations to proteins involved in excitation-contraction coupling, the impacts of multi-day NO_3_^−^ consumption may be significant for NO bioavailability [60]. For instance, acute NO_3_^−^consumption may result in signaling effects that change the function of myofibrillar proteins [61], but NO_3_^−^loading over several days may provide further enhancements due to increased NO_3_^−^content stored in skeletal muscles [62]. Supporting these findings, Wong et al. [12] recommended a chronic supplementation protocol with a high beetroot dosage (>12.9 mmol/day for 6 days) for high-intensity and sprint interval training. More studies are necessary to determine the appropriate dosage recommendations for NO_3_^−^supplementation and whether intermittent efforts are influenced.

### 4.4. Effects of Beetroot Intake on Endurance Exercise

Previous reports consistently suggest exhaustive tests appeared to benefit from beetroot supplementation [14,46]. However, the present systematic review revealed inconsistent results [25,26]. This fact could be related to the inter-individual variability or/and environmental conditions modulating beetroot effects. Regarding subject characteristics, individuals with lower and moderate oxidative capacities presented greater benefits from beetroot intake than individuals with a higher oxidative capacity [63]. In the present systematic review, athletes completed ~11 stages in the study conducted by Antonietto et al. [25], whereas about 15 stages were accomplished in the study of Miraftabi et al. [26]. These findings indicate the recruited athletes by Miraftabi et al. [26] had greater cardio-respiratory fitness, which blunted their benefits from beetroot intake. As a result, nitrate supplementation was suggested to increase sub-maximal effort tolerance in athletes with a high degree of training, while also raising doubts about the drop in oxygen consumption [46]. An alternative explanation is that taekwondo athletes in the study of Miraftabi et al. [26] improved exercise economy by achieving higher power output with the same O_2_ consumption [7,46]. Contrarilywise, hypoxic conditions may be present during exercise, which facilitates the conversion of nitrite to NO [4]. The lack of O_2_ in the air might theoretically be compensated by boosting blood flow to improve oxygen delivery [4]. Therefore, the inconsistent findings may be linked to variationsin the environmental conditions.

### 4.5. Effects of Beetroot Intake on Core Balance, RPE and Cognitive Performance

Balance is an important factor in combat sports performance [64]. Investigating the impact of 140 mL of beetroot juice onGreco-Roman wrestlers, Tatlici et al. [37] showed supplementation improved dynamic and static balance at rest, as well as after a fatiguing stimulus. The benefits of beetroot intake were associated with an improvement of afferent input, reduction of the delay in reaction time, and improvement in proprioceptive acuity [37]. These findings suggest beetroot juice is a useful strategy to maintain balance performance and prevent sports injuries in combat sports. Balance is crucial in combat sports, especially in grappling contests (e.g., judo, ju-jitsu, wrestling) [64], and further investigations are required to generalize these findings for females and athletes with different competitive levels and backgrounds.

It is well known that higher blood flow to the brain’s frontal lobe, which is responsible for motor control and decision-making, is one potential mechanism explaining beetroot’s effects on RPE [65]. Decreased blood flow to the brain during training is known to be a major cause of fatigue [66]. Therefore, increased cerebral blood flow can play a role in reducing RPE and improving function after beetroot supplementation [65]. However, in the present systematic review, studies did not report a significant impact of beetroot intake on RPE, independent of exercise. The unchanged RPE following the ingestion of the beetroot supplement may be caused by a drop in central motor command as a result of the exercise’s maintenance of contractility [67]. This may be due to the fact that RPE displays a central feedback process in which a motor order output is dispatched from the motor zones to the sensory brain, allowing conscious awareness of the actions relevant to motor yield [65].

Regarding beetroot benefits on cognitive performance, Miraftabi et al. [26] showed that, even without affecting maximal oxygen consumption, beetroot juice intake (i.e., 400 mg of NO_3_^−^) improved cognitive function after the PSTT. These positive effects on cognition may arise from the positive effect of NO on neurovascular coupling [68] and increased cerebral perfusion, primarily in the prefrontal cortex responsible for executive function [69]. Therefore, NO_3_^−^ may help to slow the deterioration of cognitive function during competition, especially in reaction time [70].

### 4.6. Physiological Basis behind Beetroot Effects

The beetroot pathway mediates several physiological effects influencing skeletal muscle contraction [2,7]. The generated performance enhancement following beetroot intake was generally attributed to an improvement in the efficiency of mitochondrial respiration [71] and oxidative phosphorylation [72]. This is evident in combat sports as beetroot supplementation increased muscle O_2_saturation during exercise recovery and decreased blood lactate levels after isokinetic exercise (i.e., lactate clearance improvements) [38]. This result may be related to blood flow and O_2_transport improvements [2,7]. Yet, the effects of beetroot supplementation on muscle oxygenation are inconsistent. The chronic (i.e., eight days) effect of supplementation with 100 g of beetroot-based nutritional gel increased forearm muscle O_2_ saturation during exercise recovery [38]. Conversely, the acute effect of the same supplementation protocol did not induce similar effects in another investigation [41]. This indicates the effects of supplementation on muscle O_2_saturation are cumulative and supplementation duration may modulate beetroot effects [12,62]. 

The improvement in O_2_ kinetics can minimize the metabolic disturbance in muscles brought on by the build-up of metabolites accumulated during non-oxidative metabolism [73]. Especially, beetroot may reduce the cost of ATP and alter intramuscular substrates and metabolic generation associated with muscular fatigue [54], resulting in abridged lactate generation [74]. However, findings revealed beetroot supplementation did not affect [bLa-] as compared to placebo during intermittent exercise [26]. These results were inconsistent with those reported by Wylie et al. [57], which showed that beetroot increased [bLa-] across 24 × 6 s and 7 × 30 s sprints protocols, but not in the 6 × 60 s protocol. This suggests beetroot benefits were modulated by the effort-to-pause ratio. The increases in O_2_consumption may be accounted for by nitrate propensity for oxidative energy metabolism [16]. An increase in NO_3_^−^ levels have been proposed to enhance NO availability with subsequent better oxygen transport due to vasodilation [7]. Although this has been associated with, but not proven in the lab, such an effect would favorably affect phosphocreatine resynthesis by optimizing the phosphocreatine system in the mitochondria [16]. This could be evident as the two studies [25,26] on endurance performance showed [bLa-] did not change, even without improvements in O_2_ consumption.

The main effect of beetroot supplementation on the cardiovascular system is associated with the vasodilator capacity of NO [75]. In fact, the NO vasodilatation effect results in a higher fraction of oxygenated hemoglobin in muscle, as well as a lower rate of whole-body oxygen uptake [7]. In combat sports, during isometric and isokinetic muscle contractions, chronic or acute beetroot juice supplementation resulted in the upholding of blood pressure [40] and blood volume [38,41]. Moreover, during a specific endurance exercise, Miraftabi et al. [26] noted that beetroot supplementation did not affect HR responses to exercise [26]. Due to their high-intensity training schedule, combat sports athletes may develop physiological adaptation in the conduit artery (i.e., increased basal artery diameter), which may reduce NO’s influence on vasodilatation during exercise [76]. 

### 4.7. Studies’ Limitations and Perspectives

All included studies presented good methodologies as assessed by the Cochrane Risk of Bias tool; however, there are some methodological issues that need be addressed. Interestingly, among the included studies, only Antonietto et al. [25] performed an a priori power analysis. Consequently, the lack of ergogenic potential in the case of some investigations [26,39,42] may be related to the lack of power calculation. Thus, conducting an a priori power analysis for the primary outcome is recommended in future investigations. Additionally, there is a dearth of studies including female combat sports athletes, which makes these findings difficult to generalize to females. It is believed that further studies including both sexes are required to further understand the effects of beetroot supplementation. Moreover, even with the introduction of an ergogenic supplement, combat sports performance can also be affected by additional technical and psychological aspects [25]. Therefore, further studies on the effects of beetroot supplementation on technical-tactical and psychological performances are warranted. Furthermore, research on the impact of NO_3_^−^on the brain is still in its infancy [70], thus, the effect of beetroot supplementation on brain activity (e.g., encephalography) could be the subject of further studies. Additionally, well-trained athletes have increased daily energy expenditure and often an enriched diet, when compared to the general population [63]. Therefore, the limited effects of beetroot supplementation on combat sports athletes could be related to higher nitrate dietary intake, preventing them from experiencing additional positive effects. Hence, evaluating blood nitrate concentration before and after beetroot supplementation may be utilized as a control mechanism. Additionally, among the nine included studies, no one has tested the success of blinding; future investigations should consider the effectiveness of blinding. Furthermore, given the optimal dose of beetroot is presented as mmol of NO_3_^−^ [12], it is recommended to express the concentration of NO_3_^−^found in beetroot as mmol. This is required given the large variations in the NO_3_^−^ content of different beta vulgaris varieties [77].

According to the American College of Sports Medicine [78], adequate selection of nutrients and supplements, adjusting intake according to the exercise performed, is necessary for optimal performance in athletes. Incidentally, the tests have to be feasible for acquiring relevant physiological mechanistic data and to sufficiently induce the correct metabolic demand for the sports discipline of interest [79]. Accordingly, using a more specific testing procedure could be an important issue, as specificity could modulate athletes’ motivation to exercise and reflect real sports demands. Finally, the ergogenic potential of NO_3_^−^ supplementation is largely dependent on the reduction of concentrated NO_3_^−^to NO_2_^−^ [18], which is regulated by anaerobic bacteria in the oral cavity [11,46]. Although the oral microbiome may be disturbed by many oral substances (e.g., antibiotics, antibacterial mouthwash, gum chewing) [46], only five studies (i.e., 55% of studies) [36,37,39,40,42] reported controlled environments for the oral microbiome (i.e., refrain from mouthwash). Presumably, this may be the result of variability in oral nitrate reductase activity to reduce nitrate to nitrite [11]. Therefore, athletes should refrain from mouthwash usage when evaluating nitrate supplementation benefits.

### 4.8. Review Strengths and Limitations

This is the first systematic review addressing the effects of beetroot supplementation on combat sports outcomes. This paper presented a comprehensive revelation of the available investigations and an objective criticism of its methodologies. Moreover, the study was conducted according to the updated PRISMA guidelines, without any restriction on language or year. Additionally, the critical points and perspectives issued from this study can help to draw further strong investigations into this topic. Conversely, certain limitations could be acknowledged in this systematic review. Firstly, the present findings are limited to young male athletes. Additionally, there were some difficulties in the interpretation of the actual impact of beetroot intake due to the varied design protocols of studies. Likewise, considering the variety of measured outcomes and the small number of studies per outcome, performing a meta-analysis may not be a reasonable approach for providing convincing evidence about intervention effects [80]. It was previously reported that, in small meta-analyses, the heterogeneity statistic *I*^2^ can be biased [81], and statistical methods for possible detection of publication bias are underpowered [82]. Moreover, an optimal dose cannot currently be recommended, as factors such as the level of physical fitness, exercise modality (e.g., explosive, intermittent, endurance), and duration (among others) may modulate beetroot effects.

## 5. Conclusions

A single dose or multiday usage of beetroot supplementation could be an effective strategy for combat sports athletes to improve various aspects of performance. Specifically, beetroot supplementation improved oxidative metabolism and muscle force production during isometric and isokinetic exercise, which could be of great importance, especially for grappling combat sports practitioners. The discrepancy across studies appeared to relate to the inter-individual variability (e.g., training level,), dosing protocol (e.g., chronic or acute), mode of NO_3_^−^delivery and amount, muscle group activated (i.e., upper vs. lower body), exercise mode, and test duration. More studies are needed to elucidate the impacts of manipulating dosing regimens in various exercise modalities as well as within female athletes to understand how and when to administer beetroot to enhance combat sports performances.

## Figures and Tables

**Table 1:** Search strategy on each database.

Database	Terms Combination
Pubmed	((“Nitrates”[MeSH] OR “nitrate*”[All Fields]) OR (“Nitrites”[MeSH] OR “nitrite*”[All Fields]) OR “beet*”[All Fields]) AND (“combat sports” [All Fields] OR “Martial Arts”[Mesh] OR “judo*” [All Fields] OR “taekwondo*” [All Fields] OR “Wrestling”[Mesh] OR “wrestl*” [All Fields] OR jiu-jitsu [All Fields])
Web of Science	(Nitrates OR nitrate OR Nitrites OR nitrite OR beetroot OR beet OR beets OR “beta vulgaris” OR chard OR chards) AND (“martial arts” OR “combat sports” OR judo OR judoka OR judokas OR judoist OR judoists OR taekwondo OR taekwondoist OR taekwondoists OR wrestling OR wrestler OR wrestlers OR jiu-jitsu)
Scopus	(nitrate* OR nitrite* OR beet*) AND (“martial arts” OR “combat sports” OR judo* OR taekwondo* OR wrestl* OR jiu-jitsu)
Cochrane Library	(nitrate* OR nitrite* OR beet*) AND (“martial arts” OR “combat sports” OR judo* OR taekwondo* OR wrestl* OR jiu-jitsu)
Scielo	(Nitrates OR nitrate OR Nitrites OR nitrite OR beetroot OR beet OR beets OR “beta vulgaris” OR chard OR chards) AND (“martial arts” OR “combat sports” OR judo OR judoka OR judokas OR judoist OR judoists OR taekwondo OR taekwondoist OR taekwondoists OR wrestling OR wrestler OR wrestlers OR jiu-jitsu)
SportDiscuss	(nitrate* OR nitrite* OR beet*) AND (“combat sports” OR “martial arts” OR judo* OR taekwondo* OR wrestl* OR jiu-jitsu)

**Table 2:** Characteristics of the population investigated in different studies are presented as mean (SD).

Study	Sex	Body Mass (kg)	Age (years)	Experience (years)	Training Regime	Country
Tatlici [36]	M	76.75 (5.4)	21.87 (2.3)	NR	3 days per week	Turkey
Antonietto et al. [25]	M	77.8 (11.7)	26.8 (8.8)	NR	≥3 days per week	Brazil
Tatlici et al. [37]	M	76.75 (5.4)	21.87 (2.3)	NR	3 days per week	Turkey
de Oliveira et al. [38]	M	81.3 (10.1)	29 (9)	NR	≥15 h per week (3 to 5 times)	Brazil
Miraftabi et al. [26]	M	64.8 (4.0)	20 (4.0)	>5	5 times per week	Iran
Bernardi et al. [39]	NR	79.29 (10.07)	24.9 (4.6)	2.9 (1.6)	5 days per week	Brazil
Khosravi et al. [40]	M	66.4 (9.2)	19.2 (1.6)	9.5 (2.8)	NR	Iran
de Oliveira et al. [41]	M	79.72 (10.09)	29.92 (8.5)	>5	15 h per week	Brazil
Tatlici and Cakmakci [42]	M	76.66 (19.37)	23 (2.28)	10.5 (0.5)	NR	Turkey

M: male; F: female; NR: not reported.

**Table 3:** Summary of the studies examining the impacts of beetroot supplementation on combat sports performance.

Study	Design	Sample Size	Timing	Doses	Form	Duration	Measures	Results
Tatlici [36]	DBRCD	Eight Greco-Roman wrestlers	150 min	140 mL (NR as mmol of NO_3_^−^)	Juice	Acute	knee extensions and flexion at 60° Shoulder internal and external rotator isokinetic strength test at 60°	↑ In peak torque of shoulder internal (*p* = 0.048) and external (*p* = 0.024) rotator values. ↔ In the knee strength (*p* > 0.05).
Antonietto et al. [25]	DBRCD	12 Taekwondo athletes	NR	1 g Beetroot extract (NR as mmol of NO_3_^−^)	Capsule	Acute	PSTT [bLa-], VO_2max_	↑ Absolute VO_2Peak_ (*p* = 0.048), absolute VO_2max_ atventilatory threshold (*p* = 0.044) and complete stages (*p* = 0.009) ↔ In [bLa-] (*p* = 0.46)
Tatlici et al. [37]	DBRCD	Eight Greco-Roman wrestlers	150 min	140 mL (NR as mmol of NO_3_^−^)	Juice	Acute	Dynamic and Static Balance testing before and after fatiguing exercise	At rest: ↑ in static MLSI (*p* < 0.001), dynamic OSI (*p* = 0.03) and APSI (*p* = 0.01). At fatigue: ↑ in static OSI (*p* < 0.001), APSI (*p* = 0.01), dynamic OSI (*p* = 0.01), APSI (*p* = 0.02), and MLSI (*p* = 0.02).
de Oliveira et al. [38]	DBRSD	12 Brazilian jiu-jitsu athletes	120 min	100 g (12.2 (0.2) mmol of NO_3_^−^)	Gel	Chronic (eight days)	Handgrip isometric and isotonic exercises Blood volume Muscle oxygenation	↓ In ∆MVC decline after Handgrip isometric exercise (*p* < 0.05) ↑ Forearm SmO_2_ during exercise recovery (*p* < 0.05)
Miraftabi et al. [26]	DBRCSD	Eight Taekwondo athletes	150 min	60 mL 120 mL (400 and 800 mg of NO_3_^−^)	Juice	Acute	FSKT-mult, CMJ, and the PSTT RPE; [bLa-]; HR Stroop test	↔ For PSTT, CMJ, and FSKT performances (*p* > 0.05) ↔ For [bLa-], RPE, and HR (*p* > 0.05) After the PSTT, cognitive function was ↑ in BJ-400 compared to other treatments (*p* < 0.05)
Bernardi et al. [39]	DBRCSD	10 MMA athletes	120 min	400 mL (9.3 mmol NO_3_^−^)	Juice	Acute	20 all-out 6-s sprints interspersed with 24 s of recovery (20 × 6 s)	↔ For relative PP (*p* > 0.05) ↔ For relative MP (*p* > 0.05)
Khosravi et al. [40]	DBRCSD	12 Taekwondo athletes	120–150 min	140 mL (∼12.8 mmol NO_3_^−^)	Juice	Chronic (six days)	Isokinetic knee extensions RPE Blood pressure HR	↑Knee extensor peak torque and at angular velocities of 180 in the dominant leg (*p* = 0.004) and at 360°/s in the non-dominant (*p* = 0.036) ↑ In peak torque during 50 maximal knee extensions at 180°/s (*p* = 0.046) ↔In HR and blood pressure (*p* > 0.05)
de Oliveira et al. [41]	DBRCSD	14 Brazilian combat sports athletes	120 min	100 g (12.2 (0.2) mmol NO_3_^−^)	Gel	Acute	Handgrip isotonic exercise Muscle O_2_ saturation Blood volume Before and after a fatiguing exercise	↓ The decline of handgrip strength after a fatiguing exercise (*p* = 0.036) ↔ In muscle O_2_ saturation parameters, blood volume and exercise time until fatigue (*p* > 0.05)
Tatlici and Cakmakci [42]	SBRCSD	Eight Boxers	150 min	2 mL/kg of body body mass(NR as mmol of NO_3_^−^)	Juice	Acute	30-s all-out Wingate test HR; [bLa-]	↔ In [bLa-] and HR (both *p* > 0.05) ↓ In PP (*p* = 0.02), relative PP (*p* = 0.01), MP and relative MP (both *p* = 0.02)

↓: decrease; ↑: increase; ↔: No difference; APSI: anterior–posterior stability index; [bLa-]: blood lactate concentration; CMJ: countermovement jump; DBRCSD: Double-blind randomized cross-over study design; DBRSD: Double blind randomized study design; FSKT-mult: multiple frequency speed of kick Test; HR: heart rate; MLSI: medial-lateral stability index; MMA: mixed martial arts; MP: mean power; MVC: maximal voluntary contraction; NR: Not reported; O_2:_ oxygen; OSI: overall stability index; PP: peak power; PSTT: progressive specific taekwondo test; RPE: rating of perceived exertion; SBRCSD: Single- blind randomized cross-over study design; SmO_2_: muscle O_2_ saturation.

## Data Availability

Not applicable.

## Supplementary Materials

The following supporting information can be downloaded at: https://www.mdpi.com/article/10.3390/nu15020398/s1, PRISMA 2020 Main Checklist; and PRISMA Abstract Checklist; Table S1: List of excluded studies in each level.

## Registration

A priori protocol was pre-registered on the Open Science Framework (OSF) https://osf.io/85sru (accessed on 3 October 2022).
