# Supplementary material for: Does Beetroot Supplementation Improve Performance in Combat Sports Athletes? A Systematic Review of Randomized Controlled Trials
Source: Nutrients. 2023 Jan 12;15(2):398. doi: 10.3390/nu15020398 (PMC9860842; doi:10.3390/nu15020398)
Supplement: Supplementary file 1 [file nutrients-15-00398-s001.zip › nutrients-2032188-supplementary.pdf]

nutrients

MDPI

Supplementary Material

1.1.1 Table S1 | List of excluded studies in each level

Exclusion level	Studies
By title	[1-20]
By abstract	Trial Registry records: [21,22]<br>Intervention: [23-28]<br>Intervention an population: [29,30]
Full text	Intervention: [31]

References

1. Arinobu, E.; Hatsumi, A.; Hiroshi, Y.; Hisashi, O. Propagation of impulse in the heart of the beetle allomyrina dichotomus. *Comparative Biochemistry and Physiology -- Part A: Physiology* **1990**, *97*, 601-605, doi:10.1016/0300-9629(90)90134-E.
2. Bond, W.B.; Philips, T.K. Diversity, phenology, and flower hosts of anthophilous long-horned beetles (Coleoptera: Cerambycidae) in a southeastern Ohio forest. *Entomological News* **1999**, *110*, 267-278.
3. Chard, H.; Litherland, B. "Hollywood" Hulk Hogan: Stardom, Synergy, and Field Migration. *JCMS-JOURNAL OF CINEMA AND MEDIA STUDIES* **2019**, *58*, 21-44, doi:10.1353/cj.2019.0056.
4. Grgic, J.; Pedisic, Z.; Saunders, B.; Artioli, G.G.; Schoenfeld, B.J.; McKenna, M.J.; Bishop, D.J.; Kreider, R.B.; Stout, J.R.; Kalman, D.S., et al. International Society of Sports Nutrition position stand: sodium bicarbonate and exercise performance. *J Int Soc Sports Nutr* **2021**, *18*, 61, doi:10.1186/s12970-021-00458-w.

5. Hamilton, W.J.; Buskirk, R.E.; Buskirk, W.H. Social organization of the namib desert tenebrionid Beetle onymacrzs rugatzpennzs. *The Canadian Entomologist* **1976**, *108*, 305-316, doi:10.4039/Ent108305-3.
6. Havlíková, P.; Chuman, T.; Janský, B. Comparative study of fluvial lakes in floodplains of the Elbe, Lužnice and Svratka Rivers based on hydrochemical and biological approach. *Environ Monit Assess* **2017**, *189*, 639, doi:10.1007/s10661-017-6354-z.
7. Hopkins, W.G. Research for Athletes at the Prague Meeting of the European College of Sport Science. *Sportscience* **2019**, 1-15.
8. Hopkins, W.G.; Impellizzeri, F.M. Reflections on the 2010 Annual Meeting of the European College of Sport Science in Antalya, Turkey. *Sportscience* **2010**, *14*, 36-47.
9. Horton, J. Introduction: Understanding the symphony. *The Cambridge Companion to the Symphony* **2011**, 10.1017/CCO9781139021425.001, 1-12, doi:10.1017/CCO9781139021425.001.
10. Hsueh, C.F.; Wu, H.J.; Tsai, T.S.; Wu, C.L.; Chang, C.K. The Effect of Branched-Chain Amino Acids, Citrulline, and Arginine on High-Intensity Interval Performance in Young Swimmers. *NUTRIENTS* **2018**, *10*, doi:10.3390/nu10121979.
11. Johnson, K. The Return of the Geneticist: Theodosius Dobzhansky, Edward Chapin, and Museum Taxonomy. *J Hist Biol* **2022**, *55*, 443-463, doi:10.1007/s10739-022-09694-5.
12. LÓpez-Laval, I.; Mielgo-Ayuso, J.; Terrados, N.; Calleja-GonzÁlez, J. Evidence-based post exercise recovery in combat sports: a narrative review. *J Sports Med Phys Fitness* **2021**, *61*, 386-400, doi:10.23736/s0022-4707.20.11341-0.
13. MacKenzie, A. Star shooter. *Automotive Industries AI* **2001**, *181*, 43.
14. Niijima-Yaoita, F.; Nagasawa, Y.; Tsuchiya, M.; Arai, Y.; Tadano, T.; Tan-No, K. Effects of methylphenidate on the impairment of spontaneous alternation behavior in mice intermittently deprived of REM sleep. *Neurochem Int* **2016**, *100*, 128-137, doi:10.1016/j.neuint.2016.09.010.
15. Preston-Mafham, K. Resource defence mating system in two flies from Sulawesi: Gymnonerius fuscus Wiedemann and Telostylinus sp. near duplicatus Wiedemann (Diptera: Neriidae). *Journal of Natural History* **2001**, *35*, 149-156, doi:10.1080/002229301447916.

16. Rice, M.E.; Merickel, F.; Macrae, T.C. The Longhorned Beetles (Coleoptera: Cerambycidae) of Idaho. *Coleopterists Bulletin* **2017**, *71*, 667-678, doi:10.1649/0010-065X-71.4.667.
17. Shiraki, Y.; Hiruma, M.; Sugita, T.; Ikeda, S. Assessment of the treatment protocol described in the guidelines for Trichophyton tonsurans infection. *Nihon Ishinkin Gakkai Zasshi* **2008**, *49*, 27-31, doi:10.3314/jjmm.49.27.
18. Son, W.M.; Sung, K.D.; Bharath, L.P.; Choi, K.J.; Park, S.Y. Combined exercise training reduces blood pressure, arterial stiffness, and insulin resistance in obese prehypertensive adolescent girls. *Clin Exp Hypertens* **2017**, *39*, 546-552, doi:10.1080/10641963.2017.1288742.
19. Vicente-Salar, N.; Fuster-Muñoz, E.; Martínez-Rodríguez, A. Nutritional Ergogenic Aids in Combat Sports: A Systematic Review and Meta-Analysis. *Nutrients* **2022**, *14*, doi:10.3390/nu14132588.
20. Voolma, K.; Õunap, H. Diversity and abundance of insects and some other arthropods in unmanaged and managed forests. *Forestry Studies* **2006**, *44*, 95-111.
21. Irct2017072735331N. The effect of concentrated beetroot juice on muscle strength and balance in taekwondo athletes. *https://trialsearch.who.int/Trial2.aspx?TrialID=IRCT2017072735331N1* **2017**.
22. Irct20191013045088N. The Effect of Mouth Rinsing and Watermelon Juice Ingestion on Nitric Oxide, Total Antioxidant Capacity and Performance in Elite Female Taekwondo. *https://trialsearch.who.int/Trial2.aspx?TrialID=IRCT20191013045088N1* **2019**.
23. Agricola, N.P.A.; Guillo, L.A. Endothelial nitric oxide concentrations in the Saliva of Jiu-Jitsu athletes. *Revista Brasileira de Medicina do Esporte* **2020**, *26*, 298-301, doi:10.1590/1517-869220202604218318.
24. Camarço, N.F.; Sousa Neto, I.V.; Nascimento, D.C.; Almeida, J.A.; Vieira, D.C.L.; Rosa, T.S.; Pereira, G.B.; Prestes, J. Salivary nitrite content, cognition and power in Mixed Martial Arts fighters after rapid weight loss: a case study. *J Clin Transl Res* **2016**, *2*, 63-69.
25. Hakaufová, L. Sugar beet varieties registered in 2017. *Listy Cukrovarnicke a Reparske* **2017**, *133*, 51-56.

26. Mori, M.; Ishikawara, F.; Tomoda, T.; Yamada, S.; Okamoto, M.; Itabashi, H.; Seki, Y.; Matsumoto, R.; Shoho, Y.; Martha, L., et al. Use of capillary electrophoresis with dual-opposite end injection for simultaneous analysis of small ions in saliva samples from wrestlers undergoing a weight training program. *Journal of Chromatography B: Analytical Technologies in the Biomedical and Life Sciences* **2016**, *1012-1013*, 178-185, doi:10.1016/j.jchromb.2016.01.037.
27. Rennesson, S. Wrestling beetles and ecological wisdom: How insects contribute to the cosmopolitics of Northern Thailand. *Southeast Asian Studies* **2019**, *8*, 3-24, doi:10.20495/seas.8.1_3.
28. Yang, W.H.; Heine, O.; Pauly, S.; Kim, P.; Bloch, W.; Mester, J.; Grau, M. Rapid rather than gradual weight reduction impairs hemorheological parameters of Taekwondo athletes through reduction in RBC-NOS activation. *PLoS One* **2015**, *10*, e0123767, doi:10.1371/journal.pone.0123767.
29. Cubrilo, D.; Djordjevic, D.; Zivkovic, V.; Djuric, D.; Blagojevic, D.; Spasic, M.; Jakovljevic, V. Oxidative stress and nitrite dynamics under maximal load in elite athletes: relation to sport type. *Mol Cell Biochem* **2011**, *355*, 273-279, doi:10.1007/s11010-011-0864-8.
30. Cubrilo, D.; Radovanovic, D.; Cikiriz, N.; Krivokuca, R.; Milovanovic, M.; Ristic, P.; Jakovljevic, V. Comparison of nitric oxide dynamics under maximal exercise in different sports. *Medicus* **2006**, *7*, 103-106.
31. Liu, T.H.; Wu, C.L.; Chiang, C.W.; Lo, Y.W.; Tseng, H.F.; Chang, C.K. No effect of short-term arginine supplementation on nitric oxide production, metabolism and performance in intermittent exercise in athletes. *Journal of nutritional biochemistry* **2009**, *20*, 462-468, doi:10.1016/j.jnutbio.2008.05.005.
